# Innovative Hybrid Cloud Solutions for Physical Medicine and Telerehabilitation Research

**DOI:** 10.5195/ijt.2024.6635

**Published:** 2024-06-28

**Authors:** Kyrylo S. Malakhov

**Affiliations:** V. M. Glushkov Institute of Cybernetics of the National Academy of Sciences of Ukraine

**Keywords:** Cloud computing, Digital health, Telerehabilitation, Hybrid cloud environment, MedRehabBot, MedLocalGPT

## Abstract

**Purpose:**

The primary objective of this study was to develop and implement a Hybrid Cloud Environment for Telerehabilitation (HCET) to enhance patient care and research in the Physical Medicine and Rehabilitation (PM&R) domain. This environment aims to integrate advanced information and communication technologies to support both traditional in-person therapy and digital health solutions.

**Background:**

Telerehabilitation is emerging as a core component of modern healthcare, especially within the PM&R field. By applying digital health technologies, telerehabilitation provides continuous, comprehensive support for patient rehabilitation, bridging the gap between traditional therapy, and remote healthcare delivery. This study focuses on the design, and implementation of a hybrid HCET system tailored for the PM&R domain.

**Methods:**

The study involved the development of a comprehensive architectural and structural organization for the HCET, including a three-layer model (infrastructure, platform, service layers). Core components of the HCET were designed and implemented, such as the Hospital Information System (HIS) for PM&R, the MedRehabBot system, and the MedLocalGPT project. These components were integrated using advanced technologies like large language models (LLMs), word embeddings, and ontology-related approaches, along with APIs for enhanced functionality and interaction.

**Findings:**

The HCET system was successfully implemented and is operational, providing a robust platform for telerehabilitation. Key features include the MVP of the HIS for PM&R, supporting patient profile management, and rehabilitation goal tracking; the MedRehabBot and WhiteBookBot systems; and the MedLocalGPT project, which offers sophisticated querying capabilities, and access to extensive domain-specific knowledge. The system supports both Ukrainian and English languages, ensuring broad accessibility and usability.

**Interpretation:**

The practical implementation, and operation of the HCET system demonstrate its potential to transform telerehabilitation within the PM&R domain. By integrating advanced technologies, and providing comprehensive digital health solutions, the HCET enhances patient care, supports ongoing rehabilitation, and facilitates advanced research. Future work will focus on optimizing services and expanding language support to further improve the system's functionality and impact.

The advancement of modern technologies has significantly impacted intellectual activities, particularly in the realm of research and development (R&D). A notable innovation in this context is the Research and Development Workstation Environment (RDWE), an advanced iteration of automated workstations designed for contemporary research and intelligent information technologies. RDWE systems encompass the critical stages of the R&D life cycle, ranging from semantic analysis of information across various domains to the development of innovative solutions. A distinguishing characteristic of RDWE systems is their adaptability to diverse R&D activities, facilitated by the integration of multiple functional services and the capacity to incorporate new ones within a hybrid cloud environment.

The formal model, foundational principles, and development requirements for RDWE systems have been extensively examined in previous studies (O. Palagin, Kaverinsky, et al., 2023; O. V. Palagin et al., 2018, 2022). Among the most impressive examples of modern RDWE systems is the automated interactive system OntoChatGPT (O. Palagin, Kaverinskiy, et al., 2023). This system was built using advanced computational linguistics technologies, such as OpenAI's GPT-4, ontology engineering and natural language understanding support services KEn (K. S. Malakhov, Velychko, et al., 2023), and UkrVectōrēs (K. S. Malakhov, Shchurov, et al., 2023). The OntoChatGPT not only enhances human-machine interaction intuitively but also serves as a strategic tool in RDWE, driving the development of innovative information systems for scientific R&D. For an in-depth overview of the OntoChatGPT RDWE system and its evolution refer to (O. Palagin, Kaverinskiy, et al., 2023; Palagin, O.V. et al., 2024).

## Introducing the Modern Concept of Telerehabilitation in the Physical Medicine and Rehabilitation (PM&R) Domain

***Telerehabilitation*** represents the current state-of-the-art concept in the field of PM&R, integrating advanced information and communication technologies (ICT) to revolutionize the traditional rehabilitation process. This modern approach supports the rehabilitation of patients through telemedicine, digital health, in-person therapy, and quality of life assessments, providing a comprehensive and flexible framework for patient-centered care.

### Telemedicine Integration

Telerehabilitation applies telemedicine to provide remote support and monitoring for patients undergoing rehabilitation. This includes: *Virtual Consultations* (enabling healthcare professionals to conduct remote assessments and provide guidance through video conferencing); *Remote Patient Monitoring* RPM (K. S. Malakhov, 2022) (utilizing wearable devices and sensors to track patients' progress and transmit data to healthcare providers for continuous oversight); and *Digital Therapy Programs* (delivering personalized exercise regimens and therapeutic activities through mobile apps and online platforms, allowing patients to engage in their rehabilitation from home).

### Digital Health Enhancement

Incorporating ICT within digital health, telerehabilitation enhances the quality and effectiveness of in-person therapy in rehabilitation centers. This includes: *Electronic Health Records* (EHR) (streamlining the management of patient information to facilitate coordinated care among multidisciplinary teams); *Interactive Tools* (implementing virtual reality (VR) and augmented reality (AR) technologies to create immersive therapeutic experiences, dynamically adjusted to patient performance); *Integrated Telemedicine* (combining in-person sessions with remote consultations to provide continuous care and support, ensuring patients benefit from consistent therapeutic guidance).

### Traditional Rehabilitation Synergy

Telerehabilitation does not replace but rather complements traditional rehabilitation practices, emphasizing the importance of hands-on therapeutic techniques and direct patient-provider interactions. This includes: *Physical Therapy* (conducting manual therapies, exercises, and interventions to aid physical recovery); *Occupational Therapy* (facilitating activities and exercises designed to help patients regain independence in daily tasks); *Speech and Language Therapy* (offering sessions aimed at improving communication abilities and addressing swallowing disorders).

### Quality of Life Assessment

Telerehabilitation incorporates tools to evaluate the patient's overall well-being, with a specific focus on the quality of life (QOL) (V. Romanov et al., 2023; V. O. Romanov et al., 2023). This includes: *Questionnaires* (instruments designed to assess various aspects that define QOL, such as physical health, mental well-being, social interactions, and functional abilities. These questionnaires help healthcare providers understand the impact of rehabilitation on the patient's daily life and tailor interventions accordingly).

By integrating these four dimensions, *telerehabilitation* establishes a holistic and adaptive approach to patient care in the PM&R domain. This new concept ensures that:

— Patients receive continuous, tailored care regardless of their physical location, enhancing accessibility and convenience.
— Healthcare providers can utilize technology to deliver more effective and efficient rehabilitation services, improving patient outcomes.
— Rehabilitation programs become more engaging and supportive, fostering better patient compliance and satisfaction.
— The patient's quality of life is continuously monitored and assessed, allowing for adjustments to be made to optimize therapeutic outcomes.

Overall, telerehabilitation represents a significant advancement in the PM&R field, combining the strengths of telemedicine, digital health, traditional rehabilitation, and quality of life assessments to offer a versatile and patient-centered approach to rehabilitation care.

In early 2022, a research group of the Glushkov Institute of Cybernetics, including the author, led by its scientific supervisor Petro Stetsyk, became one of the winners of the “Science for Safety and Sustainable Development of Ukraine” competition held by the National Research Foundation of Ukraine. The project is titled “Development of the cloud-based platform for patient-centered telerehabilitation of oncology patients with mathematical-related modeling” (K. S. Malakhov, 2023b), aims to create an innovative cloud-based platform for the telerehabilitation of cancer patients. This platform will employ mathematical methods of system analysis, modeling, and optimization. The project's focus on oncology is motivated by the increasing cancer incidence rates in Ukraine (Yefimenko, 2023). The approach combines artificial intelligence techniques with mathematical methods to address complex problems within the chosen application domain.

The primary challenges of the project include:

— Developing a Hybrid Cloud Environment for Telerehabilitation medicine (HCET), encompassing services, platform, infrastructure, and incorporating domain-specific features and modern digitalization approaches to scientific R&D (its architectural, and technology setup).
— Creating an information-analytical subsystem (IAs) within the HCET to process data from spatially distributed network sources, continuously interacting with all relevant telerehabilitation experts. This subsystem will utilize interactive intelligent methods and tools implemented in the cloud platform and will be organized as an RDWE in computational linguistics, featuring a specialized set of services and a problem-oriented dataset.

This article discusses the general functional architecture of HCET and its technical requirements, focusing on three interacting subsystems: medical rehabilitation (MRs), information and analytical (IAs), and Telerehabilitation (TRs). It also explores the architectural and technological organization of the hybrid HCET using the RDWE system model.

## Hybrid Cloud Environment for Telerehabilitation Medicine HCET

The core technical requirements for the creation of the HCET and its underlying information technology for the telerehabilitation of cancer patients have been developed, with a comprehensive list available in the “Technical requirements for hybrid cloud platform for telerehabilitation medicine” (K. S. Malakhov & Palagin, 2024). A distinctive feature of the proposed information technology and the related HCET architecture is the integration of artificial intelligence methods with precise mathematical techniques to optimize the entire telerehabilitation process. This approach ensures a reliable assessment of the patient's condition, effective intervention strategies, optimal trajectory design for rehabilitation, prognostics, and more.

The telerehabilitation platform is implemented as a suite of services operating within an ontology-driven (Litvin et al., 2020, 2021, 2022; O. Palagin & Petrenko, 2018), service-oriented architecture (Litvin et al., 2020; A. V. Palagin, 2006; O. V. Palagin, 2016; O. V. Palagin et al., 2014). The overall functional architecture comprises two primary subsystems: the MRs, and IAs, also known as the cognitive subsystem.

**Figure 1:**
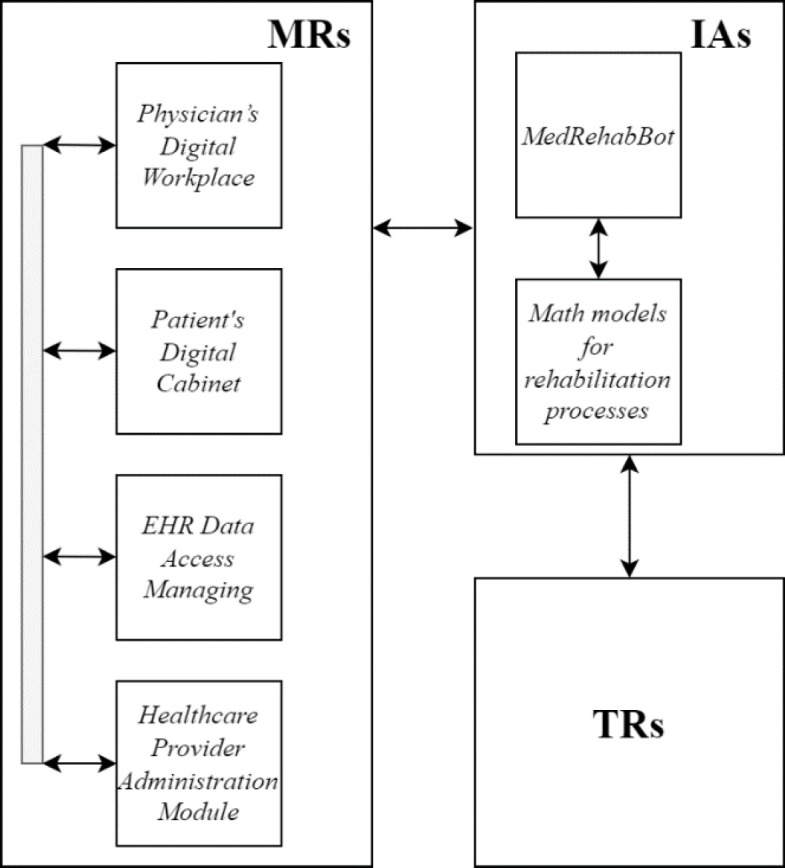
The General Functional Architecture of the HCET

The general functional architecture of the HCET is shown in Figure 1.

The MRs subsystem includes key functional modules such as:

— ***Physician's Digital Workplace***. Used by PM&R specialists, multidisciplinary rehabilitation team members, specialized healthcare physicians, and primary care physicians. This module provides a comprehensive digital interface for managing patient care and coordinating with other healthcare professionals.
— ***Patient's Digital Cabinet***. An online portal for patients to access their rehabilitation plans, track progress, and communicate with their healthcare providers. This module empowers patients by providing them with easy access to their rehabilitation information and resources.
— ***EHR Managing Module***. Handles the storage, retrieval, and management of electronic health records, ensuring secure and efficient access to patient data. This module is critical for maintaining comprehensive and up-to-date medical records.
— ***Administration and Registry Module***. Manages the administrative tasks associated with the telerehabilitation process, including scheduling, resource allocation, and documentation. Maintains a registry of patients, treatments, outcomes, and other relevant data (supports data collection and analysis, enabling the continuous improvement of rehabilitation strategies). This module ensures the smooth operation of the rehabilitation program by handling logistical and bureaucratic aspects.

These modules are essential for facilitating the telerehabilitation process according to legislative acts of Ukraine discussed in previous study (K. S. Malakhov, 2023a). A critical component is the subsystem for telemedicine support of telerehabilitation interventions, which establishes and refines the optimal trajectory of the telerehabilitation process, predicts and evaluates the effectiveness, and supports interaction among multidisciplinary rehabilitation team members.

The cognitive subsystem provides information and analytical processing of data from spatially distributed network sources. This continuous interaction with all relevant experts involved in the telerehabilitation process utilizes interactive intelligent methods and tools implemented within HCET. It is represented by the MedRehabBot component of the IAs subsystem for patient-centered telerehabilitation of cancer patients (Kaverinsky & Malakhov, 2023b; Litvin et al., 2022; O. Palagin, Kaverinsky, et al., 2023; Palagin, O.V. et al., 2024). Developed on a specialized dataset of PM&R domain (K. Malakhov, Vakulenko, et al., 2023), MedRehabBot includes web services for contextual and semantic analysis of text documents, search and classification, knowledge prediction, OWL ontology generation, and the creation of semantic trees, and graph knowledge bases within the domain.

The IAs subsystem, equipped with intelligent information and analytical support functions, evaluates the effectiveness of telerehabilitation strategies, enabling medical experts, researchers, and administrators to systematically enhance the telerehabilitation process, ensure service quality, and achieve the best outcomes for patients undergoing rehabilitation.

The functioning of the IAs subsystem is grounded in the creation of a unified general model, its precise mathematical justification, and the resolution of optimization tasks across the entire problem space. From the perspectives of project management, task distribution, and functional architecture, the HCET is represented by three interacting subsystems: MRs, IAs, and TRs.

### The Three-layer Model of the Hybrid HCET

The hybrid HCET is built using an adapted three-layer model for cloud services and cloud computing (Bhowmik, 2017). This model comprises three distinct layers:

- *Infrastructure Layer (based on the Infrastructure-as-a-Service, IaaS model)*. This layer provides the foundational level of service, enabling the management of processing and storage resources, communication networks, and other essential computational resources. It allows for the deployment and execution of arbitrary software, including operating systems (OS), application software (AS), and system utilities. The infrastructure consists of three main components: Hardware Resources (this includes servers, storage systems, client systems, and network equipment); Operating Systems and System Software (this encompasses virtualization tools, automation tools, and core resource management tools); Middleware (this software, such as virtual OS management tools, facilitates the integration and management of the infrastructure components).
- *Platform Layer (based on the Platform-as-a-Service, PaaS model)*. This layer provides access to information technology platforms, including operating systems, database management systems, middleware, and development and testing tools hosted in the cloud. The entire IT infrastructure, including computational networks and storage systems, is managed by the provider. The provider determines the available platform types and the set of configurable platform parameters. Developers are granted the ability to utilize these platforms, create virtual instances, install, develop, test, and run application software on them while dynamically adjusting the amount of consumed computational resources.
- *Service Layer (based on the Software-as-a-Service, SaaS model).* At this layer, end-users (clients) are provided access to developed services and software via a thin client (web browser) or an application programming interface (API). This layer enables the direct use of applications without the need for users to manage the underlying infrastructure or platforms.

**Figure 2:**
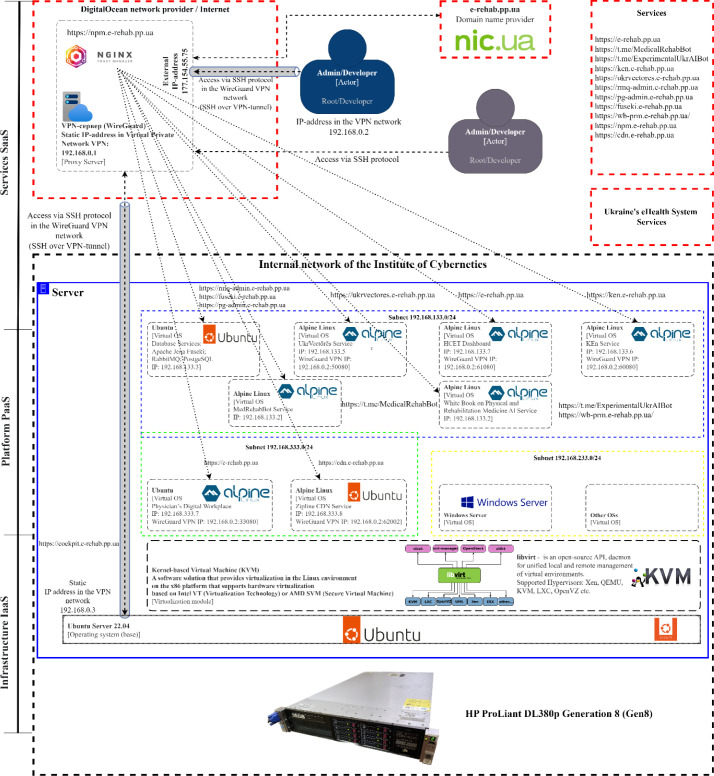
The Comprehensive Architectural and Structural Diagram of the Hybrid HCET

A comprehensive architectural and structural diagram of the hybrid HCET and its components is shown in Figure 2. The diagram includes the following components (hardware and software, external services, interfaces, and network elements):

- *HP ProLiant DL380p Gen8 Server* – the high-performance server is a key component of the Infrastructure layer of the hybrid HCET. Located in a specialized room at the Institute of Cybernetics, it ensures the reliability and high availability of the platform's services and resources. The server's technical specifications include: *CPU* – 2x Intel® Xeon® Processor E5-2695 v2, providing high performance and multitasking capabilities; *RAM* – 400 GB Advanced ECC memory, enabling efficient processing of large data volumes; *Storage* – 2x 400 GB SSDs in RAID 1 for additional reliability, and 8x 400 GB SSDs in RAID 10 for optimized speed, and durability; *Network Connection* – 1 Gbps, ensuring fast access to resources and data; *Power Supply* – 2x 460 Watt power supplies, guaranteeing uninterrupted server operation; *Uninterruptible Power Supply* – Eaton 5Cs 1500VA, protecting against power outages and ensuring equipment operation during electrical failures.
- *Base OS* – Ubuntu 22.04.3 LTS Jammy Jellyfish serves as the base OS for the server. This version of Ubuntu is known for its stability, reliability, and wide range of supported applications for workstations and servers. It is part of the Infrastructure layer of the hybrid HCET, ensuring the reliable operation and interaction of all platform components.
- *Virtualization Module based on Kernel-based Virtual Machine (KVM)* – KVM is a high-performance virtualization solution integrated directly into the Linux kernel. Designed specifically for the x86 architecture, KVM uses the capabilities of modern Intel and AMD processors that support hardware virtualization through Intel VT (Virtualization Technology) and AMD SVM (Secure Virtual Machine) technologies. A key feature of KVM is its ability to run multiple virtual machines with different operating systems on a single physical host, with each virtual machine using its own Linux kernel, and resources being efficiently and flexibly allocated through integration with the OS kernel. Infrastructure layer of the hybrid HCET.
- *Virtual Environment Management Module LibVirt* – this module, along with a set of corresponding tools, provides unified management of virtual environments, regardless of their location—locally or remotely. One of the key features of LibVirt is its versatility and flexibility: it supports a wide range of virtualization systems, including Xen, QEMU, KVM, LXC, Virtuozzo, Microsoft Hyper-V, and others. This makes LibVirt an ideal choice for administrators and developers seeking a flexible and efficient solution for managing virtualization in diverse environments. Infrastructure layer of the hybrid HCET.
- *Virtual OSs* – various specialized operating systems are used at the Platform layer of HCET. These systems play a key role in ensuring the stable and efficient operation of all platform components, including services, modules, and subsystems. The list of virtual OSs used at this level includes: Ubuntu 22.04.3 LTS Jammy Jellyfish; Alpine Linux 3.18 (a lightweight and secure operating system ideal for containers – Docker, Podman, Kubernetes); Microsoft Windows Server 2022 (a robust platform for deploying enterprise applications); Microsoft Windows 10 Pro.
- *Proxy Server / VPN Server* – An external virtual private server (VPS) that ensures the functioning of a virtual private network (VPN) using the modern WireGuard security protocol. WireGuard (Donenfeld, n.d.) is distinguished by its high level of data protection and optimized performance. Additionally, this server operates the Nginx Proxy Manager (*Nginx Proxy Manager, n.d.*), responsible for managing domains, SSL certificates, redirects, and streams. This set of tools allows for reliable, secure, and flexible access to network resources, as well as optimizing and automating web traffic management processes. Services layer of the hybrid HCET.
- *Domain Name Registrar* – the NIC.UA (Domain Registrar NIC.UA, n.d.) service is responsible for the registration and management of the cloud platform's domain name – https://e-rehab.pp.ua. Additionally, NIC.UA ensures the stability and security of subdomains used for various services, modules, and platform components. Choosing this registrar guarantees not only reliability but also ease of domain resource management, as well as the ability to quickly expand and adapt to new requirements and user needs. It should be noted that domain names in the pp.ua zone are provided free of charge in Ukraine. Services layer of the hybrid HCET.
- *Network Component of the hybrid HCET* – the network structure is based on the internal network of the Institute of Cybernetics, characterized by a high level of isolation. This closed network is integrated with the external Proxy Server/VPN Server through a reliable VPN tunnel based on WireGuard technology. This configuration allows for efficient and secure data exchange between the internal network of the Institute of Cybernetics and the external internet, ensuring the confidentiality, integrity, and availability of information.

### Development Kit Overview for Hybrid HCET

The development kit, including services and software applications at the current stage of development of the hybrid HCET, consists of the following developed services and applications.

#### Hospital Information System (HIS) Focused on the PM&R Domain

**Figure 3:**
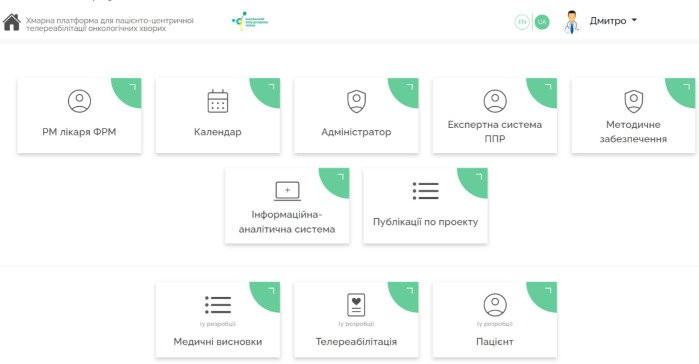
The HCET home page UI

The hybrid HCET is implemented in the form of a HIS focused on the PM&R domain (D. V. Vakulenko & Vakulenko, 2023) (in Telerehabilitation mode). The HCET home page is shown in Figure 3.

Main Components of the PM&R Physician's Digital Workplace:

- Access to Patient Profile – Includes Personal Information, Emergency Contacts, and Trusted Person Information.
- Telerehabilitation Module – encompasses the history of previous examinations and rehabilitation programs.
- Working with Rehabilitation Program Goals (D. Vakulenko et al., 2023; D. V. Vakulenko & Vakulenko, 2023) – this module allows for setting the goals of the rehabilitation program for each patient. These goals can be individualized based on the patient's condition and needs. The submodule allows for additional customization.
- Additional Examinations (via RPM) – plan additional examinations, consultations, functional tests, and conduct surveys.

The current user interface (UI) of the hybrid HCET is available in Ukrainian. This ensures that users can interact with the system in their native language, facilitating ease of use and accessibility for Ukrainian-speaking medical professionals and patients.

Future updates of the HCET will include an English language option, broadening the system's usability to a wider audience. This enhancement will cater to non-Ukrainian speaking users and foster international collaboration in the field of PM&R.

**Figure 4:**
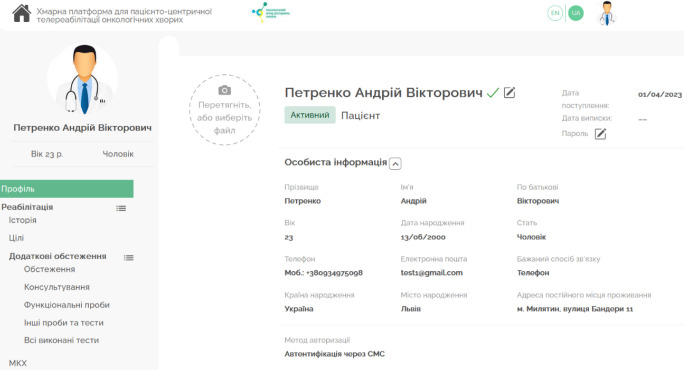
The UI of the PM&R Physician's Digital Workplace

The interface of the PM&R Physician's Digital Workplace, with submodules for Telerehabilitation, Additional Examinations, and Telerehabilitation Tools within HCET, is shown in Figure 4.

**Figure 5:**
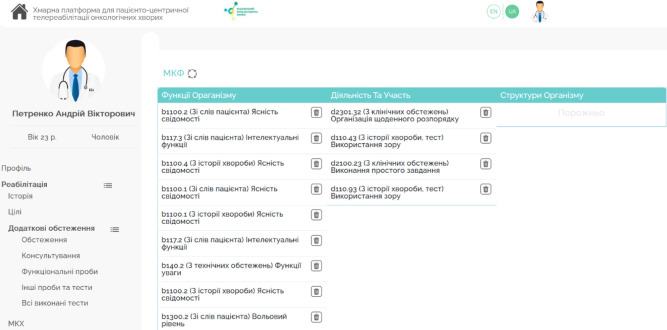
The UI of the Integration of Forms for ICPC, ICD, ICF, ICHI, and Information on Surveys and Consultations Completed

*Integration of Forms for: International Classification of Primary Care (ICPC), International Classification of Diseases (ICD), International Classification of Functioning, Disability and Health (ICF), International Classification of Health Interventions (ICHI) classifiers*. This component (shown in Figure 5) enables users to enter and view data related to the referrals and activities of the National Health Service of Ukraine, using international classifiers such as ICPC, ICD, ICF, and ICHI.

**Figure 6:**
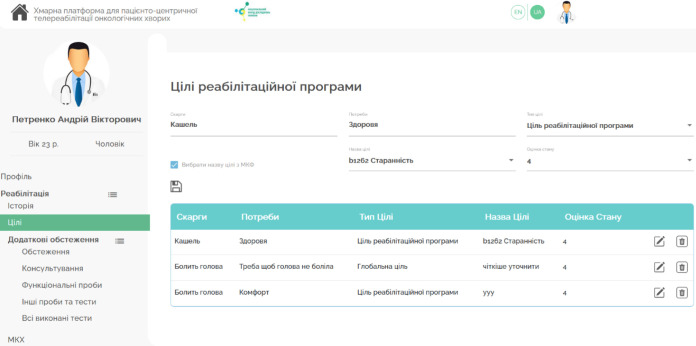
The UI of the Planning, Composition, and Evaluation of Rehabilitation Program Effectiveness Component.

*Planning, Composition, and Evaluation of Rehabilitation Program Effectiveness*. This element allows for creating rehabilitation program plans, including categorical goals, intervention tables, and lists of telerehabilitation tools, as well as evaluating the program's effectiveness (shown in Figure 6). The evaluation process can involve specialists from various fields, forming a multidisciplinary team.

*Telerehabilitation Tools Tab for Detailing Rehabilitation Program Components*. This subcategory allows for detailed specification of rehabilitation program components. It includes the following tools: Morning and Therapeutic Exercises (physical exercises that help strengthen muscles, and improve motor functions); Independent Activities (exercises or tasks that the patient can perform independently to support telerehabilitation); Massage (techniques used to improve circulation and relieve tension); Additional Motor Activity Tools (various aids, and devices that help the patient regain motor skills); Other Physical Therapy Tools (other physical methods and therapeutic approaches to improve health); Multimedia Tools (use of various media formats (images, sound, video) to address the psycho-emotional and motivational components through telerehabilitation interventions for breast cancer patients).

The entry point into the hybrid HCET is publicly available via link: https://e-rehab.pp.ua/ as a minimum viable product (MVP).

#### Natural Language Interactive Information and Reference System (with API) MedRehabBot

MedRehabBot (Kaverinsky & Malakhov, 2023a, 2023b; Palagin, O.V. et al., 2024) utilizes an information model based on a composite service represented by a three-component tuple:

- *A set of web services and application programs*. These are the fundamental software components that provide specific functionalities required for Telerehabilitation, such as data processing, user interaction, and service integration.
- *Information technology (IT) process maintenance functions*. These functions ensure the seamless operation and management of IT processes within the HCET, including system monitoring, performance optimization, and troubleshooting.
- *Elements supporting the formation of an integrated knowledge environment*. These elements facilitate the creation, management, and utilization of a comprehensive knowledge base (Kaverinsky & Malakhov, 2023a; K. Malakhov et al., 2023; O. Palagin et al., 2022; Petrenko et al., 2023), enabling effective information sharing and decision-making across the Telerehabilitation process.

To support the MedRehabBot system, the following auxiliary components were developed and enhanced.

*Conversion Utility* (Kaverinsky & Malakhov, 2023b). A software utility designed to convert the specified dataset into an ontology-related graph-based knowledge base. This knowledge base is used by the dialog system to interact with users.

*KEn Web Service* (K. S. Malakhov, Velychko, et al., 2023). A network tool and platform with an API for contextual and semantic analysis, which includes the capability to build taxonomies of documents. KEn is publicly available via link: https://ken.e-rehab.pp.ua/.

*UkrVectōrēs Web Service* (K. S. Malakhov, Shchurov, et al., 2023). A tool and platform with an API for knowledge search, classification, and prediction based on natural language understanding technologies, including distributional and semantic analysis of natural language texts. UkrVectōrēs is publicly available via link: https://ukrvectores.e-rehab.pp.ua.

*Desktop Service Suite* (Kaverinsky & Malakhov, 2023b). A set of desktop services that allow for the generation of OWL ontologies from natural language text in semi-automatic and fully automatic modes. This suite is part of the repository.

*Natural Language Phrase Analysis Web Service* (Kaverinsky & Malakhov, 2023b). A specialized web service designed to construct semantic trees for phrases, facilitating advanced semantic analysis and understanding of natural language inputs.

**Figure 7:**
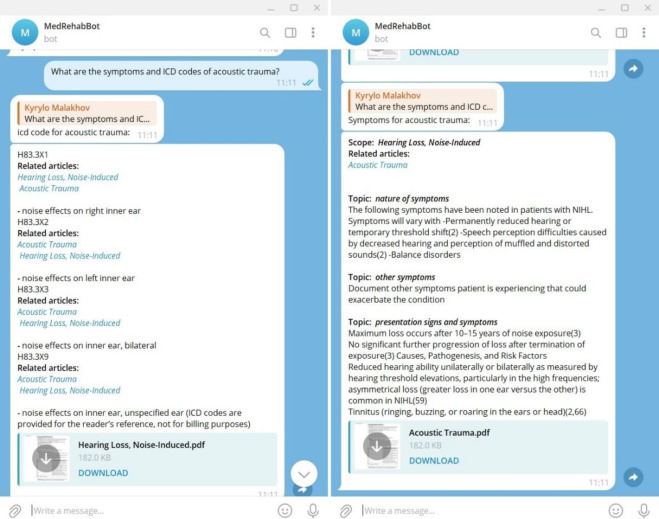
The UI of the MedRehabBot Telegram Bot

These components collectively enhance the capabilities of the MedRehabBot system, enabling sophisticated interaction, analysis, and knowledge management within the HCET framework. For the implementation of the natural language UI of the MedRehabBot system, a Telegram bot (shown in Figure 7, the MedRehabBot system supports both Ukrainian and English languages) was created. MedRehabBot is publicly available via link: https://t.me/MedicalRehabBot.

In addition to the user dialog interface, the MedRehabBot system also provides an API (MedRehabBot software platform). This allows for the integration of MedRehabBot into any HIS.

**Figure 8:**
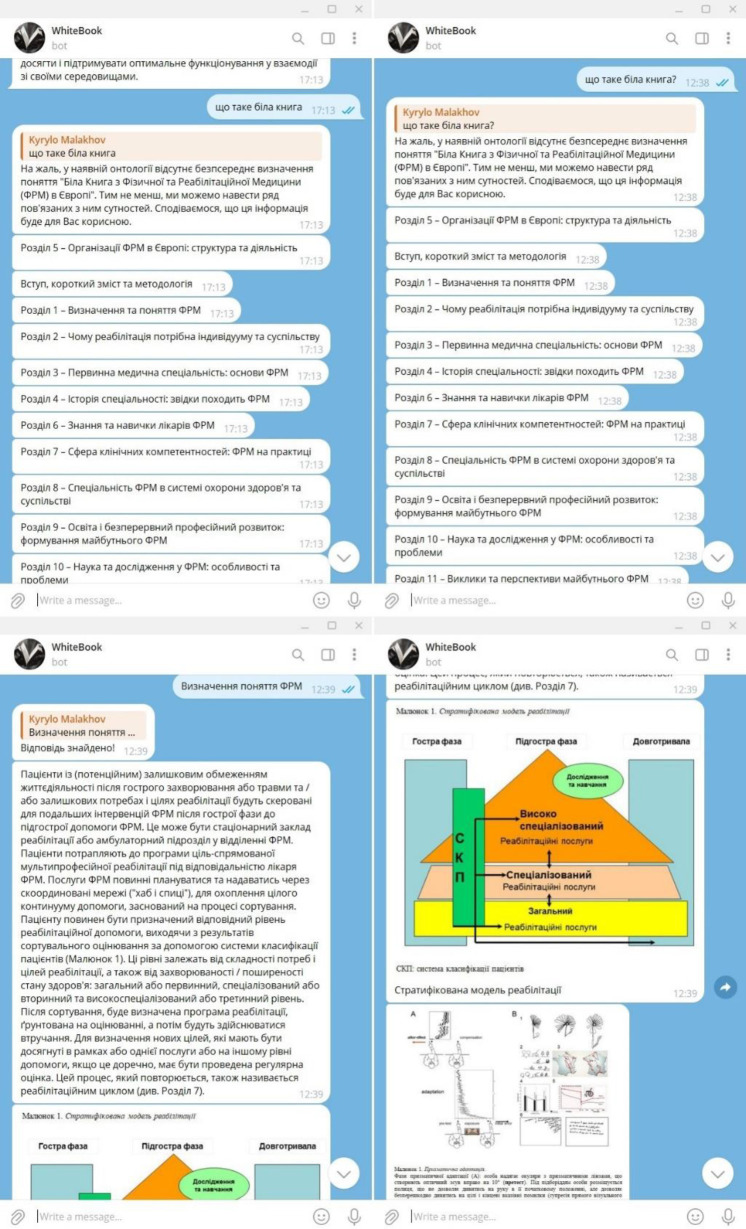
The UI of the WhiteBookBot Telegram Bot

**Figure 9:**
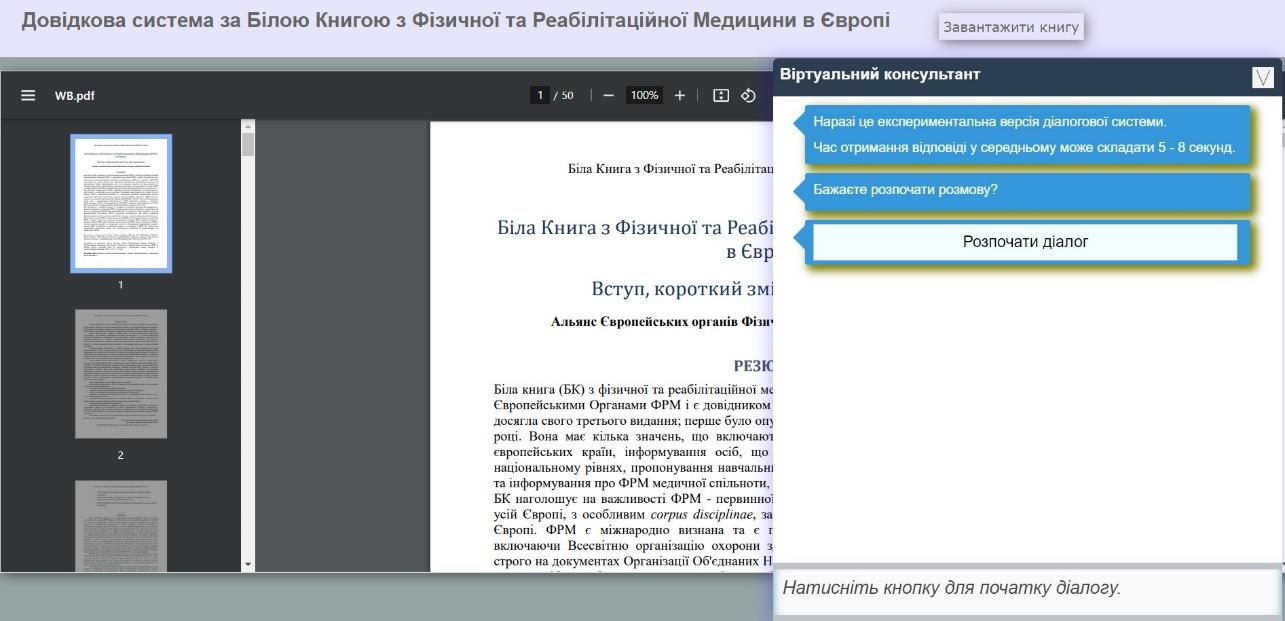
The UI of the WhiteBookBot Web Version

Using the MedRehabBot API platform technologies, a separate application, *WhiteBookBot*, has also been created. This dialog system is based on the “White Book on Physical and Rehabilitation Medicine in Europe” (European Physical and Rehabilitation Medicine Bodies Alliance, 2018; Vladymyrov, 2018), and is designed to provide users with interactive answers to questions related to physical and rehabilitation medicine. The UI of this dialog system is implemented in two parallel working versions: a Telegram bot (shown in Figure 8), and a web application (shown in Figure 9).

The web application UI is presented as a collapsible and expandable form in the bottom right corner of the screen. The main background of the page displays the contents of the PDF file of the “White Book” for viewing. This UI is currently available via link: https://wb-prm.e-rehab.pp.ua.

The UI in the form of a Telegram bot is available on the Telegram messaging service. The bot can be accessed via link: https://t.me/ExperimentalUkrAIBot. Users do not need to start a new conversation each time; this only needs to be done upon first interacting with the chatbot. After that, users can continue to input messages into the chatbot as they would in a typical Telegram conversation.

*The MedLocalGPT Project*. Special mention should be made of the MedLocalGPT project, an innovative application that integrates advanced information technology, synergizing large language models (LLMs), word embedding models, and aspects of ontology-related approaches. This includes the use of SPARQL for precise triple-storage database queries, facilitating the retrieval of fully structured, indexed data. Additionally, it employs prompt tuning to LLMs, incorporating PM&R domain-specific knowledge. The project also utilizes an improved Retrieval Augmented Generation method, enhancing the interaction between AI reference systems and end-users and improving the accuracy of information retrieval.

The MedLocalGPT project is not only a web application but also an API platform. For the current MVP stage, three API endpoints are available: Query to gpt-3.5-turbo-16k with tuning prompt (in English); Query to gpt-3.5-turbo-16k with tuning prompt (in Ukrainian); Query to PM&R domain dataset (K. Malakhov, Vakulenko, et al., 2023) with tuning prompt using gpt-3.5-turbo-16k (in English), Query to EBSCO dataset with tuning prompt using gpt-3.5-turbo-16k (in English), where the API endpoint provides users with access to a direct PDF document link that contains the knowledge related to the query.

**Figure 10:**
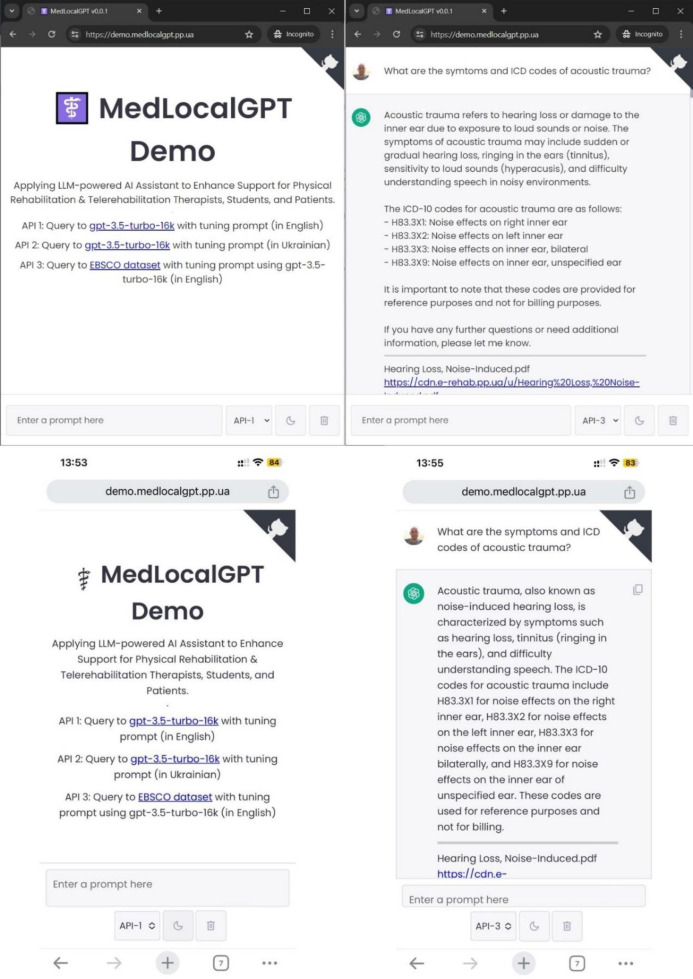
The MedLocalGPT Web Application UI (Both Desktop and Mobile Views)

The MedLocalGPT web application UI (both desktop and mobile views) is shown in Figure 10. The detailed description of all technological aspects is beyond the scope of this article and will be available in a forthcoming specific article or report.

The source code of the MedLocalGPT project is accessible through a public GitHub repository: https://github.com/knowledge-ukraine/medlocalgpt, allowing for community engagement and collaboration.

The MVP of the MedLocalGPT project is available via link: https://demo.medlocalgpt.pp.ua. Access credentials (login and password) for the MedLocalGPT web application can be obtained from the main contributor to this project, Kyrylo Malakhov, upon reasonable request.

## Conclusion

This article explored the modern concept of telerehabilitation within the Physical Medicine and Rehabilitation domain, demonstrating its transformative potential through the integration of advanced information and communication technologies. Telerehabilitation effectively bridges the gap between traditional in-person therapy and digital health solutions, providing continuous and comprehensive support for patient rehabilitation.

The general functional architecture of the Hybrid Cloud Environment for Telerehabilitation (HCET) was presented, featuring a detailed three-layer model comprising the infrastructure, platform, and application layers. Each layer is meticulously designed to support various functionalities and ensure seamless integration and scalability.

A comprehensive architectural and structural organization for the HCET was developed, encompassing the architectural setup, technological framework, and interrelation of different components. This ensures a robust and efficient system capable of meeting the demanding needs of the PM&R domain.

The core components of the HCET discussed include:

*Hospital Information System for PM&R Domain*. This MVP is tailored to the specific needs of telerehabilitation, facilitating the management of patient profiles, rehabilitation goals, and historical data through a user-friendly digital workspace for PM&R physicians.

*Natural Language Interactive Information and Reference System*. The MedRehabBot system, equipped with an API platform, supports both Ukrainian and English languages, enabling interactive, natural language processing for user queries. Additionally, the WhiteBookBot application extends this functionality by providing automated responses based on the “White Book on Physical and Rehabilitation Medicine in Europe.”

*The MedLocalGPT Project*. This innovative application integrates LLMs with domain-specific knowledge through prompt tuning. It enhances AI interactions and provides an API platform with endpoints for querying in both English and Ukrainian, as well as accessing the PM&R domain specific dataset. The project emphasizes the retrieval of structured, indexed data and offers direct PDF document links for comprehensive knowledge dissemination.

The key result of this study is the practical implementation and operation of the hybrid HCET system. By optimizing all components, and implementing bilingual support, the goal is to deploy the hybrid HCET within existing rehabilitation centers, enhancing real-world applicability and patient outcomes. This platform not only improves patient care but also facilitates advanced research in rehabilitation medicine, marking a pivotal step towards the future of digital health in PM&R.

## CRediT Authorship Contribution Statement and Important Notes

Kyrylo Malakhov (according to https://credit.niso.org): Conceptualization; Methodology; Software; Validation; Formal analysis; Resources; Investigation; Writing – Original Draft; Writing - Review & Editing.

It is important to note that the author of this article is the leading architect and developer of the comprehensive architectural and structural organization of the hybrid HCET. The author has ensured the functioning of both the infrastructure and platform layers. Additionally, the author is the main contributor to the MedLocalGPT project, and has co-authored the development of the information and analytical subsystem IAs, including the following modules: KEn Web Service, UkrVectōrēs Web Service, MedRehabBot system, OntoChatGPT system.

The mathematical-related aspects of modeling and optimization of the rehabilitation processes throughout its life cycle are beyond the scope of this article.

All other program implementations mentioned in this article have proper references to their respective authors, and developers.

## Data Availability

HCET MVP: https://e-rehab.pp.ua; KEn Web Service: https://ken.e-rehab.pp.ua; https://github.com/malakhovks/ken; UkrVectōrēs Web Service: https://ukrvectores.e-rehab.pp.ua; https://github.com/malakhovks/docsim; MedRehabBot: https://t.me/MedicalRehabBot; https://github.com/knowledge-ukraine/MedRehabBot; WhiteBookBot: https://wb-prm.e-rehab.pp.ua; https://t.me/ExperimentalUkrAIBot; MedLocalGPT: https://demo.medlocalgpt.pp.ua; https://github.com/knowledge-ukraine/medlocalgpt; OntoChatGPT: https://github.com/knowledge-ukraine/OntoChatGPT; PM&R Dataset: https://doi.org/10.5281/ZENODO.8308214.

## Further Research

The main focus of further work and research on the hybrid HCET will be on the optimization of all services and components. This includes ensuring that the platform is efficient, user-friendly, and scalable. A key aspect of this optimization will be the implementation of comprehensive Ukrainian and English language support for all services and components. This bilingual support is decisive to cater to a broader user base and enhance accessibility.

Additionally, the deployment of the hybrid HCET within an existing rehabilitation center is a significant goal. This deployment will enable real-world testing and validation of the system, allowing for the collection of valuable user feedback and performance data. Such integration will also facilitate the seamless interaction between telehealth and traditional in-person rehabilitation services, offering a more holistic approach to patient care.

Furthermore, an essential area of future research will involve enhancing the security aspects, including the user authentication process. This will be achieved using the innovative *Multilevel Face Recognition System* (Opanasenko, Fazilov, Mirzaev, et al., 2024; Opanasenko, Fazilov, Radjabov, et al., 2024), which will be integrated into hybrid HCET, MedRehabBot, and MedLocalGPT to ensure secure, and reliable access.

Further research will also explore the development of new features and enhancements based on user needs and technological advancements. This could include the incorporation of more advanced AI and machine learning algorithms to improve the personalization and effectiveness of rehabilitation programs. Moreover, expanding the dataset and refining the natural language processing capabilities will be pivotal in making the MedRehabBot and other components of the hybrid HCET more intuitive and responsive.

The ongoing collaboration with healthcare professionals, patients, and technology experts will ensure that the hybrid HCET evolves in alignment with the latest medical practices and technological innovations. This collaborative effort is essential to achieving the goal of providing a robust, reliable, and effective hybrid cloud environment for telerehabilitation medicine research and practice.

## Author Note

Kyrylo S. Malakhov

MSc in Computer Science & IT, Bachelor of Philology – English and foreign literature, Researcher, Backend developer, DevOps engineer, Microprocessor Technology Lab, V.M. Glushkov Institute of Cybernetics of the National Academy of Sciences of Ukraine. Graduate of Luhansk Taras Shevchenko National University.

Since 2023, a member of the *Interdepartmental Working Group on the Development of Telemedicine Implementation in Ukraine.*

Since 2024, a member of the *Coordination Scientific Council of the National Academy of Sciences of Ukraine on Artificial Intelligence*.
